# Proposals for consideration at IMC12 to modify provisions related solely to fungi in *Chapter F* of the *International Code of Nomenclature for algae, fungi, and plants*

**DOI:** 10.1186/s43008-024-00152-x

**Published:** 2024-08-14

**Authors:** Tom W. May, David L. Hawksworth

**Affiliations:** 1 Royal Botanic Gardens Victoria, Birdwood Avenue, Melbourne, VIC 3004 Australia; 2 Fungal Nomenclature Bureau, XII International Mycological Congress, Maastricht, The Netherlands; 3 Comparative Fungal Biology, Royal Botanic Gardens, Kew, Surrey TW9 3AE UK; 4 Department of Life Sciences, The Natural History Museum, Cromwell Road, London, SW7 5BD UK; 5 Jilin Agricultural University, Changchun, Jilin Province 130118 China; 6 Geography and Environmental Science, University of Southampton, Highfield Campus, Southampton, SO17 1BJ UK

**Keywords:** DNA types, ICNafp, Identifiers

## Abstract

Seven proposals or sets of proposals to modify the provisions of *Chapter F* of the *International Code of Nomenclature for algae, fungi, and plants* (ICNafp) have been received. These proposals are formally presented together here. The topics addressed relate to: fungi whose morph-names have the same epithet; the listing of synonyms under entries for protected names in the *Code* Appendices; the processes of protection and rejection; the use of DNA sequences as nomenclatural types; the use of genomes as nomenclatural types; and the designation of fungi known only from DNA sequences. In addition, a suggestion is included to update the mention of the World Directory of Culture Collections in Article 40.7 Note 4. A Synopsis of the formal proposals will be provided in early July 2024, and the mycological community will be invited to provide a guiding vote on the proposals with a closing date of 2 August 2024. Final decisions on the proposals will be made following debate at the Fungal Nomenclature Session of IMC12 in August 2024.

## Introduction

In the *International Code of Nomenclature for algae, fungi and plants* (ICNafp), provisions related solely to fungi are gathered in *Chapter F* of the *Code*, in which articles are numbered from F.1 onwards. While the *Code* (exclusive of *Chapter F*) is amended *via* proposals made to and decided upon by the Nomenclature Section of an International Botanical Congress (IBC), as set out in Div. III, Provision 8 of the *Code*, *Chapter F* is amended by proposals made to and decided upon by the Fungal Nomenclature Session of an International Mycological Congress (IMC) (Hawksworth et al. 2017). The current *Code* is the Shenzhen *Code* (Turland et al. 2018). *Chapter F* of the Shenzhen *Code* was amended as a result of decisions made at the Fungal Nomenclature Session of IMC11 in San Juan in 2018 and published as the San Juan *Chapter F* (May et al. 2019).

The procedure and timetable for making proposals to amend *Chapter F* was set out by May (2020). The deadline for proposals was initially advertised as 31 December 2021 but later amended to 31 December 2023, due to the shift in the timing of the next International Mycological Congress, now to be held in Maastricht, The Netherlands, on 11-15 August 2024. Several sets of proposals were submitted in December 2023 and some further proposals were accepted after that date, because they arose from work of Special-purpose Committees appointed by the previous congress or working groups of the International Commission on the Taxonomy of Fungi (ICTF).

An annotated synopsis to guide discussions will be prepared for publication in *IMA Fungus*. Due to the late publication of the proposals herein, the synopsis will be available on the IMA website ahead of publication (https://www.ima-mycology.org/index.php/formal-proposals). The guiding mail ballot about the proposals to amend *Chapter F* will close on 2 August 2024. The results of that guiding vote will be made available to the Fungal Nomenclatural Session (FNS) to be held during IMC12 on Thursday 15 August 2024, via the IMA website (https://www.ima-mycology.org/index.php/guiding-vote).

The upcoming FNS will be able to consider any suggested amendments to these sets of proposals during the Session, but no completely new proposals can now be put forward unless an exception is allowed by the Fungal Nomenclature Bureau appointed by the Congress.

In July, the XX IBC in Madrid will consider changes to the *Code* (exclusive of *Chapter F*) and the Madrid *Code* will be published in 2025. Therefore, following IMC12 in August, it is likely that there will be no separate publication of *Chapter F* and changes to *Chapter F* approved by the IMC will be incorporated directly during the preparation of the Madrid *Code*.

Note that in the series of proposals that follow, proposed new text is in **bold** type, and any deleted text in strikethrough.

## (F-001) Proposal to enable the same epithet to be retained for different morphs of the same fungus

### Background

One initially unforeseen consequence of adoption of the 1F=1N (one fungus, one name) rule in 2011, was that in instances where the same epithet had been used for the name of a newly discovered morph of the same fungus, that could be threatened by an earlier name which otherwise would have remained in synonymy.

Proposals to address this issue were developed by Hawksworth et al. (2013), and a view of mycologists attending IMC10 at Bangkok in 2014 on these was obtained through a questionnaire circulated at IMC10 in 2014; 86.9 % of respondents were in favour (Redhead et al. 2014). A formal proposal was therefore made to the Nomenclature Section meeting of the International Botanical Congress in Shenzhen in 2017 (Hawksworth 2015), at which only 16 mycologists were present. The International Commission on the Taxonomy of Fungi has found that 67% of its members supported the proposal. Views expressed at the Congress were mixed, so a Special-purpose Committee was established to consider the matter. The report of that Committee has now been published (Mitchell et al. 2024) and is to be considered along with this proposal at the IMC12 Nomenclature Session meeting in 2024.

We do not wish to reiterate the discussions of the Special-purpose Committee, but briefly acceptance of this proposal would mean that where the same epithet had been used for a newly discovered morph, that name should be treated as a new combination and not as the name of a new species, and so take its date at species rank from that of the basionym. If that is not done, as the basionym cannot now be combined into the appropriate generic name as that would make a homonym, a different epithet has to be found: that will mean either taking up the earliest other synonymized name, or if there is none introducing a completely new species name.

The argument against this approach is that the name with the familiar epithet could be safeguarded using the conservation and protected lists procedures. We find that unsatisfactory as either of those processes takes several years to be approved. In the interim, there is a danger that names with unfamiliar epithets will be introduced and confusions will occur. A clear-cut answer that can be applied immediately is ideal, especially as it is names of commoner species which tend to have most synonyms, not least ones of plant pathological or medical importance.

We note that this proposal is the converse of the provision that applied under several editions of the *Code* prior to that of 2012 (Melbourne *Code*) which ruled that where asexually typified names were used as basionyms in sexually typified genera, the intended combinations were to be treated as new species names and not new combinations, despite the clear intent of the author. There was therefore a precedent accepted in earlier editions of the *Code* for changing author citations and types for dealing with situations involving names of pleomorphic fungi.

Adoption of this proposal would mean that the type designations of the sexual morph names would lose that status, and the type would be that of the asexual morph basionym. Where necessary, the material designated as type of the sexual morph name could be re-designated as an epitype for the type of the asexual morph name (i.e. of the basionym of the combination), if (and only if) that name is “demonstrably ambiguous” (Art. 9.9).

Some examples of species whose epithets or attributions could be under threat of change if this new rule is not approved are:

1. *Ceratocystis paradoxa* (to “*C. dimorpha*”).
2. *Magnusiomyces capitatus* (to “*M. spicatum*”) discussed in Mitchell et al. (2024).
3. *Nectria ventricosa* (to “*N. cuneiformis*”).
4. *Venturia carpophila* (to “*V. amygdali*”).
5. *Yarrowia lipolytica* (to “*Y. cornealis*”).

For those wishing to recognize some groups of *Aspergillus* species at the generic level, something we do not personally support but some others continue to do (e.g. Pitt & Hocking 2022), some examples are:

(6) *Neosartorya fumigata* (to “*N. aviaria*”). One of the most cited mould species, with 180,000 mentions in Google Scholar, and which is of major medical importance.
(7) *Petromyces flavus* (to “*P. effusus*”).

We are also aware of a case where a sanctioned name is involved:

(8) *Ascodichaena rugosa* H. Butin 1977, the sexual morph of a very common fungus on *Fagus* and sometimes *Quercus* bark. The epithet was deliberately chosen to retain that of the asexual morph, *Polymorphum rugosum* (L.) D. Hawksw. & Punith. 1973. In this case the earliest epithet is *Lichen rugosus* L. 1753, which was sanctioned by Fries, and the next is *Opegrapha faginea* Pers. 1794; all typified by the asexual morph. According to the current rules, a new combination based on *Lichen rugosus* would be a later homonym of Butin’s name but the final epithet of a sanctioned name is not available for the required combination in *Ascodichaena* under Art. F.3.7. A new combination based on Persoon’s name would be required. With the present proposal, however, all that would be needed would be a change in the author citation to *Ascodichaena rugosa* (L.) H. Butin as opposed to making a homonymous combination attributed to (L.) comb. nov. and then making a conservation proposal to keep the epithet “*rugosa*”.

It is not known how many names might be affected beyond a change in author citations, but this proposal would enable them to be dealt with immediately by researchers as they are encountered without having to go through the lengthy process of proposals for conservation, rejection, or protection.

### Proposal

**(F-001) Insert a new provision in Art.F.8:**

**“** ***F.8.2*** **. If, prior to 1 January 2013, an author publishing a new species name for the morph of a fungus that had an earlier name typified by a different morph adopted the specific epithet of the name of the previously described morph, the newly published name is to be treated as a new combination and not the name of a new taxon with a different type. Designations such as “sp. nov.” and ascriptions excluding the earlier name are to be treated as formal errors requiring correction.”**

**David L. Hawksworth**^**1**^**,** **Sybren de Hoog**^**2**^**,** **John McNeill**^**3**^**, and Michael J. Wingfield**^**4**^

^1^Comparative Fungal Biology, Royal Botanic Gardens, Kew, Surrey TW9 3AE, UK;

Department of Life Sciences, The Natural History Museum, Cromwell Road, London, SW7 5BD, UK.

^2^Radboudumc-CWZ Centre of Expertise for Mycology, Nijmegen, The Netherlands

^3^Royal Botanic Garden. 20A Inverleith Row, Edinburgh EH3 2LR, UK

^4^Department of Microbiology and Plant Pathology, Forestry and Agricultural Biotechnology Institute, University of Pretoria, Pretoria 0002, South Africa

## (F-002) Proposal to clarify that a proposal to conserve a name with a conserved type does not require citation of a typification identifier

### Background

Article F.5.4 of *Chapter F* of the *Code* specifies that, from 1 Jan. 2019, new typification acts for names of fungi require citation of an identifier issued by a recognized repository, in order to be effective. In discussions in the Nomenclature Committee for Fungi it has been noted that identifiers for typifications are not provided when a proposal is published to conserve a name with a conserved type. Rather, the practice has been for one of the recognized repositories (Fungal Names, Index Fungorum, or MycoBank) to issue an identifier for a conserved type, once a proposal has been accepted, as part of the curation of the relevant name index database.

Of course, new typifications associated with conservation proposals, such as lectotypifications and epitypifications, do require citation of identifiers for the typification acts. However, the publication of a conserved type is not treated as a “designation” in contrast to the typification acts that are covered by Article 9 and 10. Hence, the requirements of Art. 7.11 (to indicate that a type is “designated”) do not apply when proposing a conserved type. A reason not to cite identifiers when proposing a conserved type is that the proposals may not be eventually recommended by the Nomenclature Committee for Fungi and/or the General Committee. As far as the issuing of identifiers, it seems useful to clarify that the proposal of a conserved type does not require citation of an identifier. Therefore, the following amendment to *Chapter F* of the *Code* is proposed.

### Proposal

**(F-002) Amend Article F.5 Note 3 as follows (new text in bold):**

“F.5.4. For purposes of priority (Art. 9.19, 9.20, and 10.5), designation of a type, on or after 1 January 2019, of the name of an organism treated as a fungus under this *Code* (Pre. 8), is achieved only if an identifier issued for the type designation by a recognized repository (Art. F.5.3) is cited.

Note 3. Art. F.5.4 applies only to the designation of lectotypes (and their equivalents under Art. 10), neotypes, and epitypes; it does not apply to the designation of a holotype when publishing the name of a new taxon, for which see Art. F.5.2**, nor does it apply to proposing a conserved type when publishing a proposal to conserve a name (Art. 14.9)**.”

**Tom W. May**^**1**^**, Luis A. Parra**^**2**^**, Marco Thines** ^**3**^**, and James C. Lendemer**^**4**^

^1^Royal Botanic Gardens Victoria, Birdwood Avenue, Melbourne, Victoria 3004, Australia

^2^Avda. Miranda do Douro 7, 5.º G, 09400 Aranda de Duero, Burgos, Spain

^3^Senckenberg Biodiversity and Climate Research Centre, Senckenberganlage 25, 60325 Frankfurt am Main, Germany; Goethe University, Department of Biological Sciences, Institute of Ecology, Evolution and Diversity, Max-von-Laue-Str. 9, 60436 Frankfurt am Main, Germany

^4^Department of Botany, Research and Collections, CEC 3140, The New York State Museum, 222 Madison Ave., Albany, NY 12230, USA

## (F-003–F-004) Proposals to remove the listing of synonyms from entries for protected names in the appendices to the code and to clarify the processes of protection and rejection

### Background

Article F.2.1 of *Chapter F* of the *International Code of Nomenclature for algae, fungi, and plants* (*Code*), dealing with protected names, states that “Protected names … which become part of the Appendices of the *Code* … once reviewed and approved … are to be listed with their types” (May et al. 2019). Lists of names proposed for protection under Art. F.2.1 have been prepared by official “working groups” (May 2017). Several lists of names proposed for protection have been recommended to be accepted by the Nomenclature Committee for Fungi (May 2017) and by the General Committee and, after ratification at IBC XIX, have been listed in the current version of the Appendices to the *Code* (Wiersema et al. 2018 on). Further lists (and additions to lists) of names proposed for protection have been recommended to be accepted in recent reports of the Nomenclature Committee for Fungi (May 2024a, b) and by the General Committee and will be considered for ratification by the Nomenclature Section of IBC XX.

All protected names that have been put forward to date cite at least one name against which they are protected. However, protected names are “treated as conserved against any competing listed or unlisted synonyms or homonyms (including sanctioned names)”. Since being protected, some further earlier names have been identified that threaten protected names, but by virtue of being protected (rather than conserved) there is no need to formally add further synonyms.

In some cases, such as the proposed addition of further earlier synonyms against which *Holwaya* Sacc. and *Neonectria* Wollenw. are protected, the additional synonyms have been formally proposed by a Working Group (May 2024a). In other cases, additional earlier synonyms have come to light, but not been formally proposed by a Working Group, but merely noted in a report of the Nomenclature Committee for Fungi, such as for *Desmidiospora* Thaxt. 1891 as a further synonym of the protected name *Ophiocordyceps* Petch 1931 (May 2024a). There is potential for further synonyms to have been established, but not noted by Working Groups or in reports of the NCF. It is not clear how additional synonyms that come to light outside of the formal process of preparing lists for protection will be added to the *Code* Appendices, under the current wording of Art. F.2.1, or if this is necessary at all.

In a few cases, some potentially earlier names have been included in proposals to protect names, because the names in question were published in the same year as the name proposed for protection, but the relative dating of the protected name and the synonyms has not been established. These synonyms have been included in the relevant Appendix to the *Code *“pending confirmation of their relative date of publication”.

These various situations call into question the need to list any synonyms of protected names. In addition, the current wording of the *Code* means that should further earlier synonyms of protected names be identified, there is no need to formally propose them for addition to the Appendices because the protected names remain protected whether or not synonyms are listed. This will lead to the situation where protected names do not necessarily include a full listing of all known or indeed any synonyms. Another issue about the listing of synonyms is the work involved in creating the Appendix entries because in some cases there are multiple synonyms. For example, as a case with numerous prior synonyms, for the protected name *Ophiocordyceps*, there are already eight generic names listed as synonyms. Taking all this into account, it is proposed that names listed for protection are listed without synonyms.

Future implications are that if mass lists of names are proposed, such as all species names within a genus that are in current use, or all names of genera within a family that are in current use, there will be no need to exhaustively determine if there are prior synonyms. Indeed, especially in groups of fungi of particular economic or medical significance, names without known synonyms could be proposed for protection, to guard against the future possibility of replacement by newly determined prior synonyms – a situation that greatly contributes to nomenclatural stability.

It is important to remember that the *Code* rules on nomenclature, not on taxonomy. The purpose of the Appendices is not to lay out a full synonymy but merely to record that certain names are protected, conserved, or rejected. Therefore, those wishing to know what are the currently accepted synonyms of a protected name should consult taxonomic treatments and databases.

Of course, conservation can over-ride protection, and if circumstances arise where a case can be made to take up an earlier name over a protected name, or there are competing names both of which are protected, a conservation proposal can be submitted. If successful, the Appendix entry would include the particular name against which the name was conserved (in line with existing entries for conserved names).

It is proposed to remove the words “listed or unlisted” as applied to competing synonyms or homonyms with the express intent that the relevant Appendices of the *Code* need only list the protected names — which nevertheless remain protected against “any competing synonyms or homonyms”. It should be noted that in cases where a conserved name is also protected, the proposed wording does not over-ride the need to list rejected names against such conserved names.

The opportunity is also taken to suggest a change to the procedures set out in Art F.2.1 and Art. F.7.1. to reflect the reality of the way that lists of names for protection or rejection have been processed. In practice, the steps have been establishment of a subcommittee (in practice called a “working group”) followed by submission of a list created by that subcommittee to the Nomenclature Committee for Fungi. Even though the role of the General Committee in initially accepting the lists has been removed, the General Committee retains a key role, alongside the Nomenclature Committee for Fungi, in approving names proposed for protection, and is also to be consulted when subcommittees are set up. We also clarify that a given subcommittee can include in lists names for protection and/or rejection and move the cross references between Art F.2.1 and F.7.1 to the new clause about the purpose of the subcommittees.

The meticulous work of John Wiersema in adding the entries for protected names to the *Code* Appendices is acknowledged. Should the proposal to amend Art. F.2.1 be accepted, the entries for protected names in the *Code* Appendix can be revised to remove the synonyms that are currently included (that arose from previous lists prepared under Art. F.2.1.).

### Proposals

**(F-003) Amend Art. F.2.1 as follows and add a new Note (new text in bold, deleted text in strikethrough):**

“*F.2.1.* In the interest of nomenclatural stability, for organisms treated as fungi, lists of names proposed for protection may be submitted to the General Committee, which will refer them to the Nomenclature Committee for Fungi (see Div. III Prov. 2.2, 7.9, and 7.10) for examination by subcommittees **may be **established by that **the Nomenclature **Committee **for Fungi (see Div. III Prov. 7.2) **in consultation with the General Committee and appropriate international bodies **for the purpose of preparing lists of names proposed for protection and/or rejection (see Art. F.7.1)for submission to the General Committee (see Div. III Prov. 2.2, 7.9, and 7.10)**. Protected names on these lists, which become part of the Appendices of the *Code* (see App. IIA, III, and IV) once reviewed and approved by the Nomenclature Committee for Fungi and the General Committee (see Art. 14.15 and Rec. 14A.1), are to be listed with their types and are treated as conserved against any competing listed or unlisted synonyms or homonyms (including sanctioned names), although conservation under Art. 14 overrides this protection. The lists of protected names remain open for revision through the procedures described in this Article (see also Art. F.7.1).”

**“*Note 1*. Names in lists of names proposed for protection may be proposed with or without the listing of synonyms.”**

**(F-004) Amend Art. F.7.1 as follows (new text in bold, deleted text in strikethrough):**

“*F.7.1.* In the interest of nomenclatural stability, for organisms treated as fungi, lists of names proposed for rejection may be submitted to the General Committee, which will refer them to the Nomenclature Committee for Fungi (see Div. III Prov. 2.2, 7.9, and 7.10) for examination by subcommittees **may be **established by that **the Nomenclature **Committee **forFungi(see Div. III Prov. 7.2) **in consultation with the General Committee and appropriate international bodies **for the purpose of preparing lists of names proposed for protection (see Art. F.2.1) and/or rejection for submission to the General Committee (see Div. III Prov. 2.2, 7.9, and 7.10)**. **Rejected n**Names on these lists, which become part of the Appendices of the *Code* once reviewed and approved by the Nomenclature Committee for Fungi and the General Committee (see Art. 56.3 and Rec. 56A.1), are to be treated as rejected under Art. 56.1, except that they may become eligible for use by conservation under Art. 14 (see also Art. F.2.1).”

Because rejected names are rejected outright there is no need to amend the way they are to appear in the *Code* Appendices (i.e. there are no synonyms). The particular Appendix to which names rejected under Art F.7.1 should be added is not specified. Should any names be rejected under Art F.7.1 it is noted that they cannot be added to Appendix V without some qualification because, unlike names already listed in Appendix V, names rejected under Art. F.7.1 “may become eligible for conservation”.

**Tom W. May** ^**1**^

^1^Royal Botanic Gardens Victoria, Birdwood Avenue, Melbourne, Victoria 3004, Australia

## (F-005) Proposal to allow the naming of fungi from DNA sequences as types

### Background

The Special-purpose Committee (SPC) on DNA Sequences as Types for Fungi was established at the XI International Mycological Congress in San Juan with a remit to investigate the use of DNA sequences as types for fungi (May et al. 2018). At present, while DNA characters are often used in describing or diagnosing new species of fungi, if no physical specimen is available, it is not possible to use a DNA sequence as the type. The need to allow formal naming of fungi known from DNA sequences alone has been discussed in a variety of publications, most recently by Wu et al. (2019), Lücking et al. (2021) and Nilsson et al. (2023). Essentially, there is a vast and increasing number of fungi known only from DNA sequences and many appear to be un-culturable microfungi, where it is simply not feasible to secure conventional type specimens, in the form of physical specimens or cultures. A few species have already been described solely from DNA (de Beer et al. 2016; Kalsoom Khan et al. 2020). These names based on DNA types are invalid under the current wording of the *Code*, but serve as examples that it is already feasible to establish names of fungi in this way using rigorous scientific practices.

The “San Juan DNA SPC” discussed the issues around fungi known only from DNA sequences and considered the outcomes of the parallel Special-purpose Committee on DNA Sequences as Types established at the Shenzhen International Botanical Congress (Thiele et al. 2023a). Two sets of proposals have been published by the “Shenzhen DNA SPC” for consideration by the Nomenclature Section of the XX International Botanical Congress to be held in Madrid in July 2024 (Thiele et al. 2023b, c). One set, 329–338, proposes to “permit DNA sequences to serve as types of names in prescribed circumstances” (Thiele et al. 2023b) and the other set, 339–348, proposes to “permit DNA sequences to be used for fixing the application of names in prescribed circumstances” (Thiele et al. 2023c). Note that the Thiele et al. (2023b, c) proposals are written to cover all groups covered under the *Code*, i.e. algae, fungi and plants, although they are intended “not [to] apply to macroscopic organisms such as vascular plants, bryophytes, macro-fungi and macro-algae …”.

The “San Juan DNA SPC” considered the issue solely in relation to fungi. Members of the SPC supportive of naming fungi known only from DNA sequences considered that the approach of using DNA as a type was preferred, rather than using DNA for “fixing the application of names” because the latter approach requires a significant alteration to the existing Principle II of the *Code* that states “The application of names of taxonomic groups is determined by means of nomenclatural types”.

The set of proposals presented below have been developed from the Thiele et al. (2023b) proposals, and are written as additions to *Chapter F* of the *Code*, the provisions applying solely to names of organisms treated as fungi. For some of the proposals by Thiele et al. (2023b), the wording has been adopted directly; but for other proposals, modifications have been made. Significant departures from the Thiele et al. (2023b) proposals include: (1) for names of fungi based on DNA as a type, a diagnosis is mandatory [the Thiele et al. (2023b) proposals allow for a DNA sequence to act as a description], (2) the diagnosis of novel species of fungi based on DNA alone must be in English, rather than English or Latin as specified for other kinds of types [a restriction not made by Thiele et al. (2023b)], and (3) there are no modifications to the requirements for effective publication [because Thiele et al. (2023b) introduced new wording that “if the type of a name of a new taxon is a DNA sequence, the sequence itself is treated as a description or diagnosis.” they needed to ensure that deposition of a sequence in a repository was not disallowed under Art. 29.1]. These modifications make for a simpler set of proposals.

Many existing species of fungi are described with information about DNA sequences, and indeed, in some cases the information about sequences is the only descriptive or diagnostic information that is presented in the protologue. The use of DNA characters alone to describe and/or diagnose is allowable under the current *Code*, which does not specify what kinds of characters are to be used in preparing descriptions or diagnoses, beyond stating that the characters cannot be “purely aesthetic features, economic, medicinal or culinary use, cultural significance, cultivation techniques, geographical origin, or geological age” (Art. 38.3). The proposals below do not change that situation, but are specifically about extending the kind of type material that may be used for fungi, to allow DNA sequences as types.

In essence, acceptance of the proposals below will mean that a DNA sequence that is lodged in an approved repository (examples of potential repositories are GenBank and the European Nucleotide Archive) can be cited as the holotype of a new species of fungus (via the repository identifier), as long as there is a diagnosis (in English) and that diagnosis is published in an approved journal (an example of a potential journal is *IMA Fungus*) along with a justification as to why it was not possible to secure a culture or a specimen as the holotype.

We are grateful for the work of the Special-purpose Committee on DNA Sequences as Types (Shenzhen) and in particular to Kevin Thiele for helpful discussions, and also to the Special-purpose Committee on DNA Sequences as Types for Fungi (San Juan) for their collegial discussions and suggestions.

### Proposal

**(F-005) Introduce a new Section X in** ***Chapter F*** **“DNA sequences as types”, with the following new articles and notes:**

**“*F.X.1.*** **For organisms treated as fungi, on or after 1 January 2026, the holotype (Art. 9.1) may be a DNA sequence (see Art. F.X.2) if, and only if, preservation of a physical specimen or isolation and maintenance of a pure culture (preserved in a metabolically inactive state) is technically unfeasible.”**

**“*Note x***. **For the purposes of Art. F.X.1, preservation is regarded as technically unfeasible if, and only if, physical specimens or pure cultures cannot reasonably be obtained using technologies available at the time of publication. Preservation is not considered unfeasible if a specimen or pure culture could not be obtained merely for reasons of inconvenience, lack of access or facilities, or if a specimen or culture was lost or otherwise not collected or isolated when it could have been.”**

The intent of this new Article and Note is to restrict the scope of DNA typification for fungi to cases where conventional typification using specimens or cultures is not technically feasible — in order to be minimally disruptive to established practice for fungi that can be conventionally typified. The wording adds a new kind of type to Article 9.1., a DNA sequence. The wording about what is and is not technically unfeasible is taken from the amendments to Art. 40.5 in Thiele et al. (2023b) Proposal 335.

It will be necessary to add a cross-reference in Art. 40.5. Because such a cross-reference is an amendment to the *Code* rather than to *Chapter F*, we cannot propose this formally here. However, this kind of cross-reference (and those other cross-references mentioned below) could be added editorially when the Madrid *Code* is prepared.

**“*F.X.2.*** **For organisms treated as fungi, in order to be validly published (see also Art. 39.2) a name of a new taxon introduced with a DNA sequence as a holotype (Art. F.X.1) must be accompanied by both** ***(1)*** **citation of an identifier issued for the holotype sequence by a recognized online repository (see Art. F.X.5(a) and App. X) and (2) a diagnosis that compares informative portions of the holotype sequence against comparable sequences of inferred phylogenetic relatives. The citation in (1) and the specification in (2) must be in English.”**

This new article establishes an alternative to the current requirement of Art. 39.2 for a Latin or English description or diagnosis or a reference (see Art. 38.13) to a previously and effectively published Latin or English description or diagnosis. There is no provision in this new article for reference to previously published diagnoses — the citation of the sequence identifier and the provision of the diagnosis must be in the one publication.

It will be necessary to add cross-references in Art. 38, such as in the list of exceptions in Art. 38.1(a).

**“*F.X.3.*** **For organisms treated as fungi that have a DNA sequence as a holotype (Art. F.X.1), an epitype (Art. 9.9) may be a DNA sequence. In order to effectively designate an epitype that is a DNA sequence, the identifier issued for the epitype sequence by a recognized online repository (Art. F.X.5(a) and App. X) must be cited, and** ***(b)*** **a diagnosis that compares informative portions of the epitype sequence against comparable sequences of inferred phylogenetic relatives must be provided.”**

This new article establishes an alternative to the current provisions concerning epitypes in Art. 9.9 (definition of epitype) and Art. 9.21 (designation of epitype). A circumstance where an epitype would be required is where two or more sequences identical to the holotype sequence are considered (from longer read sequencing) to potentially belong to different species.

Note that, following Thiele et al. (2023b), sequences may only be holotypes or epitypes (not lectotypes or neotypes). Epitypification using a DNA sequence would only be available for names originally typified using a DNA sequence. DNA sequences cannot be used for lectotypification and DNA sequences cannot be isotypes, syntypes or paratypes. Similarly, neotypification remains relevant only when an existing type specimen or illustration has been lost, so the replacement can and should be a specimen or illustration.

It will be necessary to add a note to Art. 9 to indicate that use of DNA as a type is limited to holotypes and epitypes.

**“*F.X.4*. In order to be validly published with a DNA sequence as type (see Art. F.X.1), in addition to meeting the requirements of Art. F.X.2 a name must** ***(a)*** **be published in an approved journal (see App. Y, Art. F.X.5(b)) and** ***(b)*** **be accompanied in the protologue by** ***(1)*** **a statement as to why it is believed that the taxon is new and unnamed, and** ***(2)*** **an explanation of why it was not feasible for a type specimen to be isolated, cultured, or otherwise prepared.”**

**“*F.X.5.*** **The Nomenclature Committee for Fungi in consultation with the General Committee, after seeking advice from relevant specialist committees and international societies, has the power to** ***(a)*** **appoint one or more localized or decentralized, open and accessible electronic repositories to issue the identifiers required by Art. F.X.2 and F.X.3 (see App. X),** ***(b)*** **ratify a list of approved journals for valid publication of names with DNA sequences as types (see App. Y), and** ***(c)*** **cancel or alter such appointments or ratifications at its discretion.”**

Articles F.X.4 and F.X.5 have the effect of restricting the range of journals that can be used to publish new names typified using DNA sequences. While such a restriction has been discussed in the past but not effected for names in general (such as in relation to peer-review), the SPC felt that the new provisions proposed here to allow DNA sequences as types would benefit from such a restriction. The wording of F.X.4 and F.X.5 is closely based on proposal 336 of Thiele et al. (2023b), which similarly mandates a list of approved journals.

Restricting the range of journals means that there is no need to prescribe in the *Code* the properties of DNA sequences (such as source, length etc.) needed to meet quality requirements for typification, leaving such matters, in effect, to the editors and peer reviewers of these designated journals (chosen and managed by the Nomenclature Committee for Fungi in consultation with the General Committee as specified in Art. F.X.5(b)). The SPC considered that quality assurance is a scientific issue and is best dealt with in this manner.

Requiring justification from the author(s) of a new name as to why a DNA sequence may be appropriately used to typify the new name (F.X.4(b)(1)) is intended to discourage authors from, for example, erecting a new species based solely on a novel DNA sequence as a type in a genus that includes species that have not yet been sequenced (and hence could be the source of the sequence). Requiring justification for the impracticability of obtaining a type specimen (F.X.4.(b)(2)) is intended to discourage authors from, for example, erecting a new species that is likely to be macroscopic and/or capable of being isolated or cultured.

**“*F.X.6.*** **The responsibility of** ***(a)*** **maintaining a list of approved repositories for storing sequences and issuing sequence identifiers (Art. F.X.5(a)), and** **(*b*)** **maintaining a list of approved journals for valid publication of names with DNA sequences as types (Art. F.X.4(a) and F.X.5(b)) rests with the Nomenclature Committee for Fungi (Div. III Prov. 7.1(g)).”**

This article is based on Proposal 338 of Thiele et al. (2023b), but altered so that it is the Nomenclature Committee for Fungi rather than the General Committee that has responsibility for maintaining the list of repositories and the list of journals.

**David S. Hibbett** ^**1**^ **, R. Henrik Nilsson** ^**2**^ **, Johannes Z. Groenewald** ^**3**^ **, Heather E. Hallen-Adams** ^**4**^ **, James C. Lendemer** ^**5**^ **,** **Chayanard Phukhamsakda** ^**6**^ **, Anna Rosling** ^**7**^ **, Marco Thines** ^**8**^ **, and Tom W. May** ^**9**^

^1^Biology Department, Clark University, Worcester, Massachusetts 01610 USA

^2^Gothenburg Global Biodiversity Centre, Department of Biological and Environmental Sciences, University of Gothenburg, Gothenburg 40530, Sweden

^3^Westerdijk Fungal Biodiversity Institute, Uppsalaan 8, 3584 CT, Utrecht, The Netherlands

^4^Department of Food Science and Technology, University of Nebraska-Lincoln, Lincoln, Nebraska 68588, USA

^5^Department of Botany, Research & Collections, CEC 3140, The New York State Museum, 222 Madison Ave., Albany, NY 12230

^6^Center of Excellence in Fungal Research, Mae Fah Luang University, Chiang Rai, 57100, Thailand

^7^Department of Ecology and Genetics, Uppsala University, 752 36 Uppsala, Sweden

^8^Senckenberg Biodiversity and Climate Research Centre and Goethe University Frankfurt am Main, Senckenberganlage 25, 60325 Frankfurt am Main, Germany

^9^Royal Botanic Gardens Victoria, Birdwood Ave, Melbourne, Victoria 3004, Australia

## (F-006) Proposal to allow genomic sequences to serve as types of names of organisms treated as fungi

### Background

Environmental sequencing has revolutionised the discovery of new lineages of microscopic organisms, in particular bacteria, protists, and fungi (O’Brien et al. 2005; Geisen 2016; Seppey et al. 2017; Nilsson et al. 2019). Because of the overwhelming amount of potentially new taxa hiding behind the operational taxonomic units (OTUs), and the realisation that the huge amount of potentially new species would take decades or centuries to describe, there were calls to facilitate the description of new species based on environmental sequencing (De Beer et al. 2016; Lücking and Hawksworth 2018), which were refuted by others (Thines et al. 2018; Zamora et al. 2018).

While an overwhelming majority of mycologists were against a formal naming of species based on single gene sequences, as evidenced by the lack of support of previous proposals to enable the typification of names by sequence data, it seems useful that environmental sequences would be given citable identifiers, similar to GenBank numbers, protein codes, or deep space objects. But, such a naming system for OTUs based on short sequences needs to be quite different from giving a definite species name, due to the uncertainty associated with such names, as discussed previously (Thines et al. 2018; Zamora et al. 2018).

However, there is also an irrefutable benefit in allowing genomic sequence data to serve as a type. This applies to organisms that cannot be cultured and documented in classical ways, which then could have a type different from an illustration, which is currently the only possibility for typification of this “dark matter” of biodiversity (James et al. 2020; Lücking et al. 2021). However, an illustration of such an organism has two notable shortcomings. First, the organism has to be imaged first, which is difficult to achieve for organisms detected by environmental sequencing. Second, an illustration that serves as a type cannot be investigated with molecular methods to infer phylogenetic positions and distinctiveness from similar species, ultimately needing an epitype to be designated. Thus, allowing genomic sequence data as a type can provide a more reliable and stable naming system, if three basic considerations are being taken into account.

First, a type should enable an unambiguous identification so that other species can also be delineated against it. This renders it obvious that single genes are not suitable as type sequences, as in many groups of fungi and oomycetes multigene phylogenies are needed to resolve species boundaries (Kepler et al. 2014; Choi et al. 2015; Kruse et al*.* 2018; Johnston et al. 2019).

Second, in contrast to a physical type, type sequences cannot reveal information beyond the sequence information given. While it is possible to sequence even genomes of historical specimens of plant parasites to reveal additional information on species, e.g. *Phytophthora infestans* from the Irish Potato Famine era more than 150 years ago (Yoshida et al. 2013), the type sequence cannot be extended later, as there is no physical type that could be scrutinised again. Thus, it is important that the information given is sufficient for species delimitation from the very beginning.

Third, type sequences cannot be generated a second time, as the sequence information itself is the type. This is different from specimens, where ex-type sequences can be retrieved again and again from the type material. Therefore, it is of utmost importance that the sequence data are as free from artefacts as possible, or, if artefacts are not avoidable, to take care that sequences can still be used to unambiguously identify a species.

Out of the considerations above, it is evident that as much sequence data as possible should be used to serve as a type. Allowing short sequences as a type (Hawksworth et al. 2016, 2018), even with the modification by Thiele et al. (2023b), is likely to have a negative impact on nomenclatural stability and, thus, unlikely to reach the necessary majority vote to be implemented in the ICNafp. However, even though it may seem pressing to accept short sequence data as a type due to the sheer number of new lineages reported, recent advances in sequencing technologies, which enable the sequencing of a complete genome from single cells (Zahn et al. 2017; Thomé et al. 2023; Lyu et al. 2023; Seto et al. 2023) or to acquire very long sequence stretches from environmental sequencing (Tedersoo et al. 2021) highlight that this is a transitory problem. Considering, in addition, that the costs for sequencing entire genomes with long reads has dropped markedly (Pucker et al. 2022) and that the error rate in current long-read sequencing technologies has improved by more than an order of magnitude over the past five years, it does not seem to be warranted to jeopardise a stable naming of microscopic organisms difficult or impossible to cultivate for the sake of gaining a very few years of time for large scale naming of species known from environmental sequences. For every change of the ICNafp it seems to be advisable to devise them only if they take into consideration obvious future developments and are likely to be still useful after several years and decades.

In contrast to short sequences, genome sequences or larger parts of them can serve as a type, as they allow to unequivocally identify an organism, provided the three considerations above are taken into account. This would enable the naming of organisms that cannot be named in a meaningful manner under the current provisions of the ICNafp, while at the same time not overwhelming taxonomists with a flood of new names that cannot be sufficiently verified and require additional taxonomic scrutiny.

In contrast to a previous proposal by Thiele et al. (2023b), the issue of minimum requirements for a genomic sequence to be acceptable as a type is addressed in this proposal, to avoid the automated description of numerous species on the basis of sequences that cannot serve as unambiguous types, avoiding a nomenclatural nightmare. Therefore, in this proposal the typification of a name is only effected by large scale genomic sequence data that would serve the main purpose of the type — that it can be used as a touchstone to determine if a specimen belongs to a certain species or not. Consequently, the following proposals are made to change the ICNafp.

### Proposal

**(F-006) Introduce a new Section Y in** ***Chapter F*** **“Genomic sequences as types”, with the following new articles and notes:**

**“*F.Y.1.*** **For organisms treated as fungi, on or after 1 January 2026, the holotype or epitype (Art. 9.1, 9.9, 9.21, 40.5) may also be an effectively published genomic sequence (see Art. F Y.4, F Y.5) if it is technically unfeasible to preserve a specimen or pure culture preserved in a metabolically inactive state that would show the features attributed to the taxon by the author of the name or if there are technical difficulties that prevent preservation of a specimen in a way suitable for later analyses.”**

**“*Note 1.*** **For the purposes of Art. F.Y.1, preservation of a physical type for later use is technically unfeasible if there is no preservation method available that conserves diagnostic features or would allow for later nucleic acid extraction and sequence analyses with technologies available at the time of publication.”**

With this provision and the associated note, typification by a genomic sequence is restricted to cases in which otherwise illustrations would be allowed or where conventional typification is not feasible.

**“*F.Y.2.*** **For organisms treated as fungi, in order to be validly published as required by Art. 38.1, 38.2, 39.1, and 39.2 a name of a new taxon for which the type is a genomic sequence does not require a separate Latin or English diagnosis or description, or a reference to a previously and effectively published Latin or English description or diagnosis (see Art. 38.13). Instead, a statement of why it is believed that the taxon is unnamed and an explanation of why a type specimen could not be isolated, cultured, or otherwise prepared must be provided.”**

**“*Note 2.*** **For the purposes of Art. 38.1, a genomic sequence designated as the type is itself treated as a description.**

***Recommendation 1.*** **If several related species are described based on a genomic sequence as the type, authors should add a diagnosis by listing diagnostic positions in a pairwise or multiple alignment with the appropriate coordinates (e.g. Kruse &** ***al.*** **in IMA Fungus 9(1): 49–73, Table 2, Fig. 6, 2018).”**

This provision and the associated note and recommendation enable valid publication of an organism treated as a fungus in the absence of a physical type with morphological, chemical, or other descriptive features. In addition, the provision requires a rationale for the choice of a genomic sequence as type, discouraging unsubstantiated description of new taxa.

**“*F.Y.3.*** **The genomic sequence type is to be deposited in a recognized repository (App. Y) and must not be changed (but see Art. F.Y.5) and the unique identifier issued by the repository is to be cited when a name is introduced based on that sequence. In order to effect typification, the citation of the identifier issued for the genomic sequence type by a recognized repository (App. Y) is sufficient.”**

Genomic sequences permissible as types are too long to be printed, so it is reasonable to cite only the identifier to effect valid typification.

**“*F.Y.4.*** **To be permissible as a type, a genomic sequence must belong to the nuclear genome of an organism treated as a fungus.”**

This is an important addition, as mitochondria can be exchanged between species.

**“*F.Y.5.*** **A genomic sequence permissible as a type must be derived from a single sample, consist of 1 to 10,000 sequence parts (contigs) that collectively constitute the genomic sequence, and contain at least one continuous genomic sequence fragment larger than 200 kb. If later analyses establish that the genomic sequence type contains sequence data not belonging to the same species, nothospecies, or infraspecific taxon, the name remains typified by the largest genomic sequence fragment and all other sequence fragments unequivocally identifiable as belonging to the same taxon.”**

**“*Note 4.*** **A continuous sequence means a sequence without interspersed unidentified nucleotides (“Ns”) in case of assembled sequences. In case of single reads, the average read quality must exceed a Phred score of 20.”**

This provision closes the potential flood gates that would allow introduction of hundreds or thousands of names based on little information other than short sequences. At the same time, the provision allows for the naming of species from unassembled metagenomic sequencing. If this latter option is not wanted, the second sentence of the note could be omitted. The introduction of a threshold for genomic sequences to be permissible as types serves nomenclatural stability and takes into account that genome sequencing will likely soon become a standard technique in the assessment of biodiversity (Formenti et al. 2022). Introducing a quality threshold is not unusual and is also done in other articles of the code, e.g. in defining that autographs need to be done in indelible ink (Art. 30.5), a type can only be a single gathering (Art. 8.2), or that a reference to a basionym in a new combination needs to be full and direct (Art. 41.5). Thus, the introduction of a stability-promoting quality threshold is not alien to the ICNafp.

**“*F.Y.6.*** **The Nomenclature Committee for Fungi, in consultation with the General Committee, after seeking advice from relevant specialist committees and international societies as appropriate, appoints one or more open and accessible electronic repositories to issue the identifiers required by Art. F.Y.3 (see App. Z), and may cancel such appointments if the appropriate standards to issue identifiers in line with the requirements of Art F.Y.3 are not met. The Nomenclature Committee for Fungi has the responsibility to maintain a list of approved repositories.”**

**Acknowledgements**

Part of the formulations of the article changes and new articles have been taken or adapted from Thiele et al. (2023b). We are grateful to Juan Carlos Zamora as well as to members of the ICTF and NCF for constructive discussions and suggestions that led to a significant improvement of the above proposals.

**Marco Thines**^**1,2**^**, Lei Cai** ^**3**^**, Nalin Wijayawardene**^**4**^** Chayanard Phukhamsakda**^**5**^**, and Andrew N. Miller**^**6**^

^1^Goethe University Frankfurt am Main, Department of Biological Sciences, Institute of Ecology, Evolution and Diversity, Max-von-Laue-Str. 9, 60456 Frankfurt am Main, Germany

^2^Senckenberg Biodiversity and Climate Research Centre, Senckenberganlage 25, 60325 Frankfurt am Main, Germany

^3^State Key Laboratory of Mycology, Institute of Microbiology, Chinese Academy of Sciences, Beijing, 100101, China

^4^Center for Yunnan Plateau Biological Resources Protection and Utilization, College of Biological Resource and Food Engineering, Qujing Normal University, Qujing, Yunnan 655011, China

^5^Center of Excellence in Fungal Research, Mae Fah Luang University, Chiang Rai, 57100, Thailand

^6^Illinois Natural History Survey, University of Illinois Urbana-Champaign, Illinois, 61820, USA

## (F-007) Proposal to add a recommendation on the designation of fungal organisms only known from DNA sequence data

### Background

Proposals to permit DNA sequence data to be used as the types of names of fungal organisms only known from environmental sequences were made to the Shenzhen International Botanical Congress in 2017 (Hawksworth et al. 2016). The issue was debated extensively in the Nomenclature Section meetings of that Congress. The outcome was the establishment of a Special-purposes Committee on DNA Sequences as Types which has now produced a discussion paper (Thiele et al. 2023a), report (Lehtonen & Thiele 2023), and proposals for changes in the *Code* (Thiele et al. 2023b, c).

That Committee’s remit was environmental DNA sequences of all groups of organisms covered by the *Code* where conventional typification by permanently preserved material or illustrations was not possible. Two sets of essentially alternative proposals resulted: one series which would permit DNA sequences to serve as types of names in prescribed circumstances (Thiele et al. 2023b), while the other dealt with using DNA sequences to fix the application of names in prescribed circumstances but not as types (Thiele et al. 2023c).

The remit of that Special-purpose committee was all groups of organisms covered by ICNafp, so a separate proposal only covering fungal organisms was presented to IMC11 in Puerto Rico in 2018 (Hawksworth et al. 2018). There was an extensive debate in the Nomenclature Session on the issue, and a different Special-purpose Committee on DNA Sequences as Types of Fungi was established (May et al. 2018), which has still to report.

In the event that proposals of Thiele et al. (2023b) to enable DNA sequences to serve as types is not accepted at the XX International Botanical Congress in Madrid in July 2024, and no separate provision on similar lines relating only to fungal organisms is approved at the XII International Mycological Congress in Maastricht in August 2024, some interim solution will be required. If that is not the case, and the matter is left open until the next congresses in 2028 (IMC13) or 2029 (IBC XXI) there is a danger that in the meantime some authors will either disregard the *Code* or decide to follow the recently proposed SeqCode designed to deal with the same situation in prokaryotes (Hedlund et al. 2022).

As neither of those alternatives would be in the interests of maintaining the current unified system of nomenclature and registration of names across mycology, the following proposal, based on one first made to the XI International Mycological Congress by Lücking et al. (2018), merits re-consideration. It should be noted that this is equivalent to what is achieved by the use of *Candidatus* names in prokaryote nomenclature (Parker et al. 2019) but avoids prefixing binomial designations in non-italic type by “*Candidatus*”, and instead using a post-nominal.

The use of “nom. seq.” as a postnominal after a generic or binominal designation, proposed again here, has the advantage of following the established practice of using such abbreviations to indicate the nomenclatural status of names in taxonomic works; examples include nom. cons., nom. sanct., nom. inval., nom. illeg., nom. rej., nom. nov., and nom. nud.

### Proposal

**(F-007) Insert a new Recommendation and Example under Art. F.5.5:**

**“*Recommendation F.5n.*** **Identifiers can be issued by a recognized repository for sequence-based designations where there is no specimen or illustration available to serve as a nomenclatural type, but when released after effective publication such designations should have “nom. seq.”** **(*nomen sequentium*)** **appended to indicate that the designations are not validly published.**

***Ex. X.*** **The designation** ***Hawksworthiomyces sequentia*** **de Beer & al. (in Fungal Biology 120: 1332. 2016) was assigned the identifier MB815690, but as it lacks a** ***Code*** **-compliant type it is to be referred to as** ***H. sequentia*** **de Beer & al. nom. seq. or** ***H. sequentia*** **nom. seq., but not as** ***H. sequentia.*** **The designation can become available for use upon valid publication (Art. 32–45) with a** ***Code*** **-compliant type.”**

**David L. Hawksworth** ^**1**^ **, Paul M. Kirk** ^**2**^ **, and Robert Lücking** ^**3**^

^1^Comparative Fungal Biology, Royal Botanic Gardens, Kew, Surrey TW9 3AE, UK;

Department of Life Sciences, The Natural History Museum, Cromwell Road, 

London, SW7 5BD, UK.

^2^Jodrell Laboratory, Royal Botanic Gardens, Kew, Surrey TW9 3AE, UK

^3^Botanisches Garten und Botanisches Museum, Frei Universität Berin, 14195 Berlin, Germany

^3^Botanischer Garten und Botanisches Museum, Freie Universität Berlin, 14195 Berlin, Germany

## Proposal to modify the sources of abbreviations used to specify the herbarium, collection, or institution holding specimens representing nomenclatural types under the ICNapf

The following is not a proposal to amend *Chapter F*. It will not be commented on in the Synopsis nor will it be included in the Guiding vote, which is why it is un-numbered. Because it is an amendment to the *Code* (exclusive of *Chapter F*) it is a matter for the Nomenclature Section of the International Botanical Congress and the Editorial Committee for the *Code* arising from that Congress. The proposal is included here in order to provide relevant information to the Editorial Committee.

### Background

The *International Code of Nomenclature for algae, fungi, and plants* (Shenzhen *Code*; Turland et al. 2018) Article 40 specifies that the specimen representing a nomenclatural type (Art. 40.1) and the single institution in which the specimen is deposited (Art. 40.7) must be indicated. Article 40.7 Note 4 permits the institution to be abbreviated as an acronym according to Index Herbariorum (which is an online database) or the World Directory of Culture Collections. In our opinion it is important to update Note 4 to replace the World Directory of Collections of Cultures of Microorganisms with a reference to the Culture Collections Information Worldwide (CCINFO) database of WFCC-MIRCEN World Data Centre for Microorganisms (WDCM); WFCC being the World Federation of Culture Collections and MIRCEN being the Microbial Resources Centers Network.

***World Directory of Collections of Microorganisms***

Following a UNESCO sponsored meeting in 1966, a survey was undertaken by the Section on Culture Collections of the International Association of Microbiological Societies (that Section became the World Federation for Culture Collections, WFCC in 1970) to establish the first extensive global catalog of microbial resources (Sly 1996). The project had funds from WHO, UNESCO and the Commonwealth Scientific and Industrial Research Organization (CSIRO), Australia. In addition, the National Research Council of Canada and the University of Queensland (Australia) have contributed to the survey by allocating staff and material assistance (Martin and Skerman 1972).

Management of the World Directory of Collections of Cultures of Microorganisms went to the World Data Centre for Microorganisms established in 1966 as the data center of the WFCC and the Microbial Resources Centers Network (MIRCEN) under the name WFCC-MIRCEN-WDCM at the University of Queensland, Australia (Komagata 1987; Miyazaki and Sugawara 2002). After the retirement of Victor Skerman, in 1987, the WFCC-MIRCEN-WDCM was relocated to the, Institute of Physical and Chemical Research (RIKEN) in Japan, and ten years later to the National Institute of Genetics (NIG) (Komagata 1987; Miyazaki and Sugawara 2002). In 2011, WDCM moved to its present host, Institute of Microbiology, Chinese Academy of Sciences (IMCAS), Beijing, China (Wu et al. 2017).

Published in 1972, the first version of the *World Directory of Collections of Microorganisms* represented the first comprehensive list of culture collections worldwide (Martin and Skerman 1972) and included names and addresses of 329 collections in 52 countries, contact persons, and an overview of collections’ holdings. The *World Directory of Collections of Microorganisms* established the basis for future databases (Wu et al. 2017). The last version of the *World Directory of Collections of Cultures of Microorganisms* was published in 1998 and the list has since moved fully online. The information in the *World Directory of Collections of Cultures of Microorganisms* has been replaced by two online services, namely Culture Collections Information Worldwide (CCINFO) and the Global Catalog of Microorganisms (GCM) (Wu et al. 2013), for lists of collections and their holdings, respectively. Because most of the information has been moved to databases, the regular publication of the World Directory of Culture Collections has been discontinued. The two latest versions of the *World Directory of Culture Collections* were published in 2014 (Wu et al. 2017) and 2023 online through the WFCC-MIRCEN-WDCM webpage (www.wdcm.org).

***Culture Collections Information Worldwide***

CCINFO provides metadata on 846 culture collections from 79 countries and regions. The database is the major registration system and metadata archive for culture collections supported by WFCC and regional culture collection networks. WDCM assigns each collection a unique identification number and also ensures that collection acronyms stay unique. As a metadata archive, CCINFO stores detailed information about the collection, including the affiliation, funding, certification, personnel, holdings, services of culture collections (e.g., identification services, IDA), and employed preservation and quality control techniques. CCINFO provides users with an interface to search the database using keywords or a combination of several fields. In the future users will be able to distinguish actively operating collections and collections, with whom contact has been lost.

### Proposal

Art. 40.7, Note 4. Specification of the herbarium, collection, or institution may be made in an abbreviated form, e.g. as given in Index Herbariorum (http://sweetgum.nybg.org/science/ih/) or in the World directory of collections of cultures of microorganisms **Culture Collections Information Worldwide (CCID) database of the World Data Center for Microorganisms (WDCM)** **(****https://ccinfo.wdcm.org****)**.

The proposed correction to Art. 40.7 Note 4 can be done editorially and will not require any formal action but only endorsement by the Nomenclature Session at IMC12.

**Andrey Yurkov** ^**1**^ **,** **Linhuan Wu** ^**2**^ **,** **Benedetta Turchetti** ^**3**^ **, Manuela da Silva** ^**4**^ **,** **Ipek Kurtböke** ^**5**^ **, and Juncai Ma** ^**2**^

^1^Leibniz Institute DSMZ-German Collection of Microorganisms and Cell Cultures, Braunschweig, Germany

^2^Microbial resources and big data center, Institute of Microbiology, Chinese Academy of Sciences, Beijing, China

^3^Department of Agriculture, Food and Environmental Sciences, University of Perugia, Perugia, Italy

^4^Fundação Oswaldo Cruz (Fiocruz), Fiocruz COVID-19 Biobank, Rio de Janeiro, Brazil

^5^School of Science, Technology and Engineering, University of the Sunshine Coast, Maroochydore, Australia
